# Pyomyoma as a Rare Source of Postpartum Sepsis

**DOI:** 10.1155/2015/263518

**Published:** 2015-08-09

**Authors:** A. DeMaio, M. Doyle

**Affiliations:** Midlands Regional Hospital, Portlaoise, Co. Laois, Ireland

## Abstract

Pyomyoma, also known as suppurative leiomyoma, is a rare clinical complication that occurs when a leiomyoma undergoes infarction and subsequent infection. A high index of suspicion is required to make the diagnosis and can be guided by a classic triad of symptoms that includes abdominal pain, sepsis without an obvious source, and a history of leiomyoma. In the vast majority of these cases, total abdominal hysterectomy is required to avoid severe morbidity and potential mortality. We present an unusual case of a postpartum pyomyoma that was successfully treated without the need for hysterectomy. With strong clinical suspicion, early diagnosis, and appropriate management, some affected patients may preserve fertility.

## 1. Introduction

Leiomyomata are common benign tumours of the uterus with an estimated cumulative incidence of 70% for Caucasian women and greater than 80% for Afro-Caribbean women [1]. They are smooth muscle neoplasm that may lead to menorrhagia or subfertility.

Pyomyoma, or suppurative leiomyoma, is a rare complication of a leiomyoma. It results from infarction and subsequent infection of a leiomyoma. Since 1945, 16 cases of pyomyoma have been reported in the literature, including 7 related to pregnancy [2]. Total abdominal hysterectomy was the treatment undertaken in 5 of the 7 pregnancy-related cases. One additional patient was treated with laparotomy and myomectomy alone. Only one patient avoided abdominal surgery and was treated with vaginal myomectomy. Laparotomy exposes potentially critically ill patients to operative risks and hysterectomy induces infertility in a young cohort of patients.

## 2. Case Report

A gravida 3, para 2+0 Caucasian woman booked for antenatal care at 12-week gestational age. At her booking scan, a large intramural fibroid measuring 14 × 12 cm was noted. Fetal anatomical survey was normal at 22 weeks and the fibroid was noted to be above the vertex and not involving the placental site. The remainder of the pregnancy was uncomplicated. She had a spontaneous vaginal delivery at 41 weeks of gestation and was discharged with planned follow-up in gynaecology outpatient clinic 3 months later.

At 5 weeks after delivery, the patient presented with abdominal pain and was generally unwell. She was noted to have pyrexia of 39°C. Examination revealed foul-smelling vaginal discharge. Transvaginal ultrasound revealed a 9 cm intramural fibroid. There was no evidence of retained products of conception. Blood cultures were positive for anaerobes for which she received IV antibiotics. After 72 hours, she clinically improved and was discharged on oral antibiotics.

**Figure 1:**
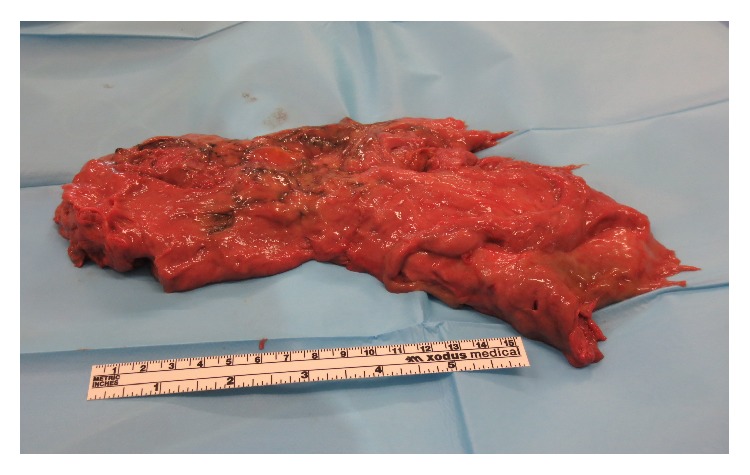
Large, malodourous mass removed manually under anaesthesia.

At 6 weeks postpartum, she re-presented complaining of the feeling of something coming down. On this occasion, she was afebrile. Examination revealed fleshy tissue protruding from the introitus. CT scan revealed a large collection in the lower segment consistent with pyomyoma. She was brought to theatre for examination under anaesthesia (EUA) and hysteroscopy. The cervical os was found to be open, and a large (27 × 10 cm), exceedingly malodourous mass was manually removed from the uterine cavity (see Figure 1).

Her postoperative course was uneventful. She was later discharged with a course of oral antibiotics. Histology of the mass revealed degenerating leiomyomata. At her follow-up visit at 6 weeks postoperatively, she reported normal menses and transvaginal ultrasound scan revealed only a small remnant fibroid.

## 3. Discussion

Since 1945, only 16 cases of pyomyoma have been reported, with 7 cases being related to pregnancy [2]. Presentation of pyomyomas ranges from painful abdominal or pelvic mass, bacteraemia of unknown origin or an acute abdomen due to rupture of the infected leiomyoma [3]. Except for 2 patients, all described cases required abdominal exploration and total abdominal hysterectomy (88%) [4–6]. One of these included a case of a pedunculated fibroid that was removed at laparotomy with minimal disturbance of the uterine body [2]. One additional case was managed with vaginal myomectomy. Ours is only the second reported case of a pyomyoma that was removed per vagina, avoiding major abdominal surgery and maintaining fertility.

These cases carry a high rate of morbidity, including sepsis and potential loss of fertility, and possible mortality [3]. A high index of suspicion is required because pyomyomas are rare. They result from infarction and infection of a leiomyoma, which may be spontaneous, or in association with diabetes, intrauterine contraceptive device, malignancy, or recent childbirth. Considering the recent increasing usage of uterine artery embolization for the management of fibroids, it is predicted that an increase in the incidence of pyomyoma will follow [7]. The differential diagnosis of pyomyoma is broad and includes any pelvic mass associated with signs of infection (pyometra, tuboovarian abscess), malignancy, degeneration of leiomyoma, or infected ectopic. A triad of known history of leiomyoma, unexplained pyrexia, and abdominal pain can be used to make the diagnosis of pyomyoma. Our case illustrates that the presence of a pyomyoma does not necessitate hysterectomy, maintaining fertility in a young woman.
